# Rapid tumor induction in zebrafish by TALEN-mediated somatic inactivation of the *retinoblastoma1* tumor suppressor *rb1*

**DOI:** 10.1038/srep13745

**Published:** 2015-09-08

**Authors:** Staci L. Solin, Heather R. Shive, Kevin D. Woolard, Jeffrey J. Essner, Maura McGrail

**Affiliations:** 1 Department of Genetics, Development and Cell Biology, Iowa State University, Ames, IA 50011, USA; 2 Department of Population Health and Pathobiology, North Carolina State University, Raleigh, NC 27607, USA; 3 Department of Pathology, Microbiology and Immunology, University of California Davis, Davis, CA 95616, USA

## Abstract

Investigating the *in vivo* role of tumor suppressor genes in cancer is technically challenging due to their essential requirement during early animal development. To address this bottleneck, we generated genetic mosaic adult zebrafish using TALEN genome editing and demonstrate somatic inactivation of the tumor suppressor *retinoblastoma1* (*rb1*) induces tumorigenesis at high frequency. 11–33% of 1-cell stage embryos injected with TALEN mRNAs targeting *rb1* exon 2 or 3 develop tumors beginning as early as 3.5 months of age. Lesions predominantly arise in the brain and show features of neuroectodermal-like and glial-like tumors. Mutant allele analysis is consistent with tumor initiation due to somatic inactivation of *rb1*, revealing a conserved role for *rb1* in tumor suppression across vertebrates. In contrast to genetic mosaics, heterozygous *rb1−*/+ adults show no evidence of neoplasia, while homozygous mutant *rb1−/−* are larval lethal. This is the first demonstration that somatic inactivation of a tumor suppressor causes cancer in zebrafish, and highlights the utility of site-specific nucleases to create genetic mosaic zebrafish for tumor suppressor gene discovery. Somatic inactivation with site-directed nucleases in zebrafish presents a rapid and scalable strategy to study tumor suppressor gene function in cancer.

Targeted somatic inactivation of tumor suppressor genes in genetic mosaic animals enables investigation of their roles in carcinogenesis if they are embryonic lethal. Mouse and zebrafish models of human cancer frequently require crosses with multiple transgenic lines to create conditional gene knockouts in predisposed mutant backgrounds^1^,^2^,^3^,^4^. Genome editing nucleases are emerging as a highly efficient alternative strategy for inactivating gene function in somatic tissues. TAL effector nucleases (TALENs) were first used for somatic gene inactivation to study vascular system development in zebrafish embryos^5^. Adult phenotypes have also been created in zebrafish and mice with CRISPR/Cas9 nuclease genome editing. In zebrafish, tissue specific CRISPR/Cas9s have been used for targeted gene knockout in the hematopoietic system^6^. Nuclease targeted somatic inactivation was successfully used in adult mice to investigate cooperating genes during liver and lung carcinogenesis^7^,^8^. Most recently, malignant brain tumor models were developed using CRISPR/Cas9 somatic inactivation of multiple tumors suppressors in embryonic and neonatal mouse brain^9^. Multiplex gene targeting with site directed nucleases simplifies the process of creating complex genotypes that normally require numerous genetic crosses. In addition, targeting nucleases allow for introduction of a range of loss of function alleles in addition to complete gene knockouts^10^,^11^.

The central role of the retinoblastoma tumor suppressor Rb1 in human cancer is well established. The Rb protein family’s main tumor suppressive role is in regulation of cell cycle entry through interaction with E2F transcription factors^12^. Rb1 function also intersects with pathways controlling cellular senescence, apoptosis, DNA repair, and chromosome integrity^13^,^14^. Mutation or alteration of the Rb1 pathway is detected in the majority of human adult gliomas^15^,^16^. The role of Rb1 in specific cancers has been studied in mice by germline mutation^17^,^18^ and somatic inactivation with conditional or tissue specific knockouts^19^,^20^. Targeted and conditional knockout of *Rb1* in combination with *tp53*, *PTEN*, or *Nf1* tumor suppressors in astrocytes and neural progenitors in adult mice induces malignant brain tumors^21^,^22^,^23^,^24^,^25^,^26^, consistent with the predicted role for these genes in preventing cellular transformation in progenitor cell populations in the brain. Combinatorial deletion of tumor suppressors with or without *Rb1* was shown to influence brain tumor phenotypes, indicating genetic disruption of cell cycle regulation in progenitors favors formation of primitive neuroectodermal tumors over glioma^26^.

Here we present a proof of principle study in zebrafish that shows genome editing nucleases can be used to study tumor suppressor gene function by targeted somatic inactivation. We demonstrate that TALEN-mediated targeting of *rb1* results in highly penetrant tumor formation in genetic mosaic adults. The zebrafish *rb1* tumor suppressor is an essential gene that functions in retinal neural progenitor proliferation and cell fate specification^27^. However, its role in cancer has not been previously studied in zebrafish. Heterozygous adult fish with stable germline *rb1* frameshift alleles did not develop lesions or tumors. In contrast, genetic mosaic adults develop tumors, predominantly in the brain, with varying degrees of proliferation and histological features of neuroectodermal-like or glial-like tumors. The majority of mutant *rb1* alleles in somatic, germline and brain tumor tissues were frameshift mutations. In tumor tissue only one or two frameshift alleles were present and overrepresented compared to wild type tissue. Together, the data indicate that biallelic inactivation of *rb1* in somatic tissue underlies neoplastic transformation and tumor initiation. Predisposing zebrafish to brain tumors by somatic inactivation of *rb1* with site-specific nucleases will be useful for investigating pathways cooperating in brain tumorigenesis. Nuclease gene targeting in zebrafish presents a rapid and simple method to screen potentially hundreds of candidate tumor suppressor genes that impact carcinogenesis.

## Results and Discussion

We used TALEN targeting to isolate germline mutations in the zebrafish *retinoblastoma1* (*rb1)* tumor suppressor gene. Single cell embryos were injected with 150 or 200 pg of TALEN mRNA targeting *rb1* exon 2 or 12.5 to 50 pg of TALEN mRNA targeting *rb1* exon 3 (Supplementary Figure S1). *rb1* exon 2 TALENs were highly efficient, obtaining up to 100% biallelic inactivation in 50% of injected embryos. Lower mutagenesis efficiency was observed with *rb1* exon 3 TALENs, and increased doses caused high toxicity and mortality. We screened three adults for germline transmission and recovered four new *rb1* alleles in exon 2, two frameshift deletions Δ4 and Δ7, and two in frame deletion Δ9 and Δ12 (Supplementary Figure S1). Homozygous Δ4 and Δ7 larvae failed to develop a swim bladder and died at ~7 days post fertilization, as reported previously for the *rb1* mutant *space cadet*^27^. Normal development and viability was observed in the in frame deletion Δ9 and Δ12 homozygotes.

**Figure 1:**
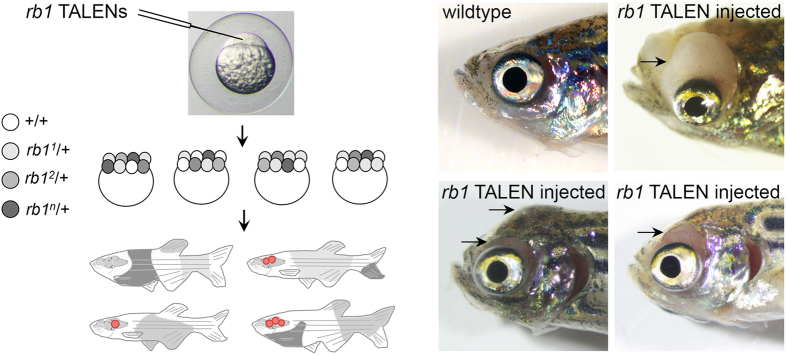
Somatic inactivation of *rb1* predisposes adult zebrafish to central nervous system tumors. (**a**) TALEN mRNAs targeting exon 2 or exon 3 of *rb1* injected into 1-cell stage zebrafish embryos induces genetic mosaicism in somatic tissue and brain tumors in adults. Mosaic embryos with variable grey shading of individual cells representing one of *n* possible *rb1* alleles. Adults with genetic mosaic tissue represented by grey areas. Red circles represent brain lesions. (**b**) Presentation of cranial tumors in three 8 month old adult zebrafish injected with *rb1* TALENs as 1-cell embryos. Age matched adult wild type fish is shown for comparison. Arrows point to masses protruding out of the cranial cavity over the eyes and distorting the dorsal head region.

During the course of this study, we observed that the genetic mosaic adults generated by somatic inactivation of *rb1* in embryos develop tumors in the head region at high frequency (Fig. 1). Beginning at 3.5 months of age, fish that were injected with *rb1* TALENs as embryos presented with tissue masses protruding out over the eyes and causing malformation of the skull (Fig. 1b, arrows). At 1.5 years post fertilization, tumor penetrance in *rb1* exon 2 and exon 3 TALEN injected fish was 33% (29/87 adults) and 11% (29/275), respectively (Table 1). The heads from 52/58 adults were dissected and revealed abnormal brain morphology disrupted by large masses. The remaining 6 adults with lesions in the head region were not further analyzed (Table 1, “unknown”). Adults often displayed abnormal swimming behavior before overt appearance of masses in the head region, providing an early indicator of brain lesions in the affected animal. In contrast, heterozygous *rb1−/*+ adults carrying either of the established *rb1* frameshift alleles do not develop tumors and are behaviorally normal.

**Figure 2:**
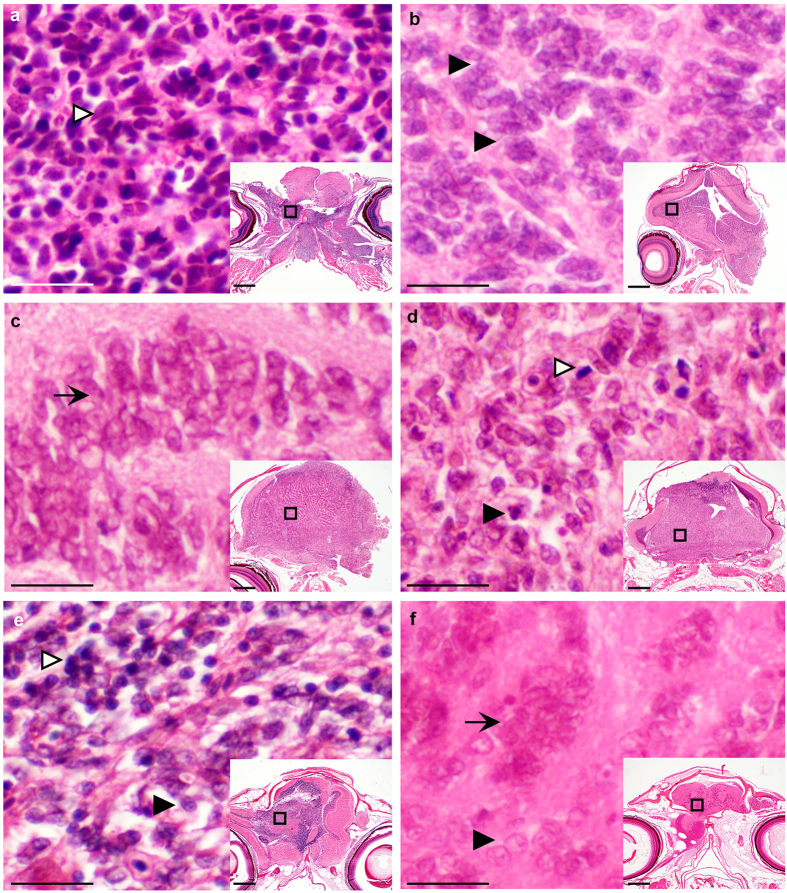
*rb1* inactivation-induced brain lesions show features of neuroectodermal-like and glial-like tumors. (**a**–**f**) Histological staining of coronal sections through heads of six adult zebrafish with brain tumors. Insets with boxed region refer to region of tumor shown in panel. (**a**) Neuroectodermal-like tumor with neoplastic cells exhibiting small hyperchromatic nuclei that form rosettes (white arrowhead). (**b**) Neuroectodermal-like tumor with neoplastic cells exhibiting oval or wedge shaped nuclei, finely granular chromatin, and occasionally forming poorly defined rosettes (black arrowheads). (**c**) Tumor with multilayered tubules (black arrow) formed from cells exhibiting oval or wedge shaped nuclei with finely granular chromatin. (**d**) Glial-like tumor with neoplastic stellate or spindloid cells exhibiting oval or irregularly shaped nuclei with granular chromatin forming poorly defined streams and bundles. Numerous mitotic figures (white arrowhead) and apoptotic cells (black arrowhead) are present. (**e**) Presumed mixed glial tumor with two cell populations. 1. Small round blue cells with hyperchromatic nuclei frequently in clusters (white arrowhead); 2. Larger cells with finely granular chromatin found in small clusters and bands (black arrowhead). (**f**) Expanded glial rests. Multiple small clusters (black arrowhead) and cellular bands of cells (black arrow) with round to oval nuclei and finely granular chromatin. no overt mass lesion. Scale bars: 200 um, inset 20 um.

Serial sections of 14/52 dissected brains in eight exon 2-targeted and six exon 3-targeted fish revealed 12 neoplastic mass lesions that infiltrated and effaced the neuropil and two brains with expanded glial rests (Table 1). The remaining dissected brains were not analyzed by histology for tumor characterization (Table 1 “not analyzed by histology)”. Most tumors exhibited numerous mitoses and variable numbers of individually apoptotic cells. Prominent vasculature was generally not a feature of individual tumors. Tumors exhibited a range of histological features (Fig. 2 and Table 1), but the majority (7/12) exhibited histological features suggestive of neuroectodermal-like tumors, including hyperchromatic or oval/wedge-shaped nuclei with rosette formation (Fig. 2a,b) or multilayered tubular structures (Fig. 2c). Four of 12 tumors contained features more consistent with glial-like tumors (Fig. 2d) or mixed glial-like tumors (Fig. 2e). The remaining two brains revealed proliferative cell populations that appeared to arise from glial rests (glial cells that are retained from embryonal neurogenesis), without formation of a clear mass lesion (Fig. 2f).

**Figure 3:**
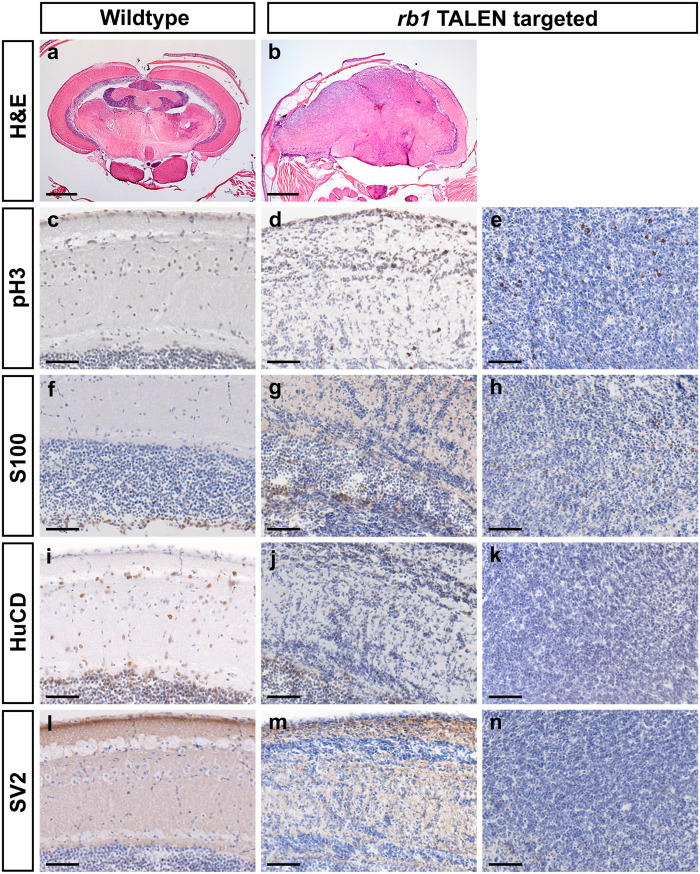
Expression of mitotic index, glial and neuronal markers in *rb1* inactivation-induced brain tumors. H and E staining of coronal sections through wild type brain (**a**) and a representative *rb1* TALEN induced brain tumor shows disruption of brain architecture (**b**). Immunohistochemical analyses on wild type (**c**,**f**,**i**,**l**) and brain tumor tissue (**d**,**e**,**g**,**h**,**j**,**k**,**m**,**n**). Phospho-histone pH3 labeling reveals regions of high mitotic index throughout the tumor (**d**,**e**). Glial marker S100 (**g**) and neuronal markers HuC/D (**j**) and SV2 (**m**) were detected within brain parenchyma infiltrated with abnormal cells. In the densely cellular regions of the tumor a few S100 positive cells were observed (**h**) while little to no expression of HuC/D (**k**) and SV2 (**n**) was detected. Scale bars in H&E panels 500 um; immunohistochemical panels 50 um.

Two brain tumors from exon 2 and exon 3 targeted fish were analyzed by immunohistochemistry for identification of proliferating cells and expression of glial and neuronal markers. Consistent with the observation of mitotic figures in hematoxylin and eosin sections (Fig. 2d), many cells expressing phospho-histone H3 were observed in different regions of each tumor (Fig. 3d,e; Supplementary Figure 2). In one brain, the overall structure appeared mostly normal, but the lateral division of the valvula cerebelli contained numerous proliferating cells not observed in wild type (Supplementary Figure 2). Images of glial and neuronal differentiation markers labeling wild type brain and a representative brain tumor are shown in Fig. 3. The glial marker S100 and neuronal markers HuC/D and SV2 were detected within less disrupted regions of the tumor positive brain including the parenchyma (Fig. 3g,j,m), which may represent non-neoplastic cells. These regions frequently showed infiltration of neoplastic cells (Fig. 3g,j,m). Expression of S100 within densely cellular regions of the tumors was limited to a few cells with similar appearance to astrocytes (Fig. 3h), although the presence of stellate astrocytes in the midbrain of adult zebrafish has not been reported^28^. Little to no expression of neuronal markers HuC/D and SV2 was observed in these tumor regions (Fig. 3k,n). The lack of expression of differentiation markers for glia (S100) and neurons (HuC/D and SV2) suggests the tumors may lack differentiated cell types, but this is not definitive. Together, the data suggest that somatic inactivation of *rb1* results in brain tumors that are highly infiltrative with diverse histological features.

**Figure 4:**
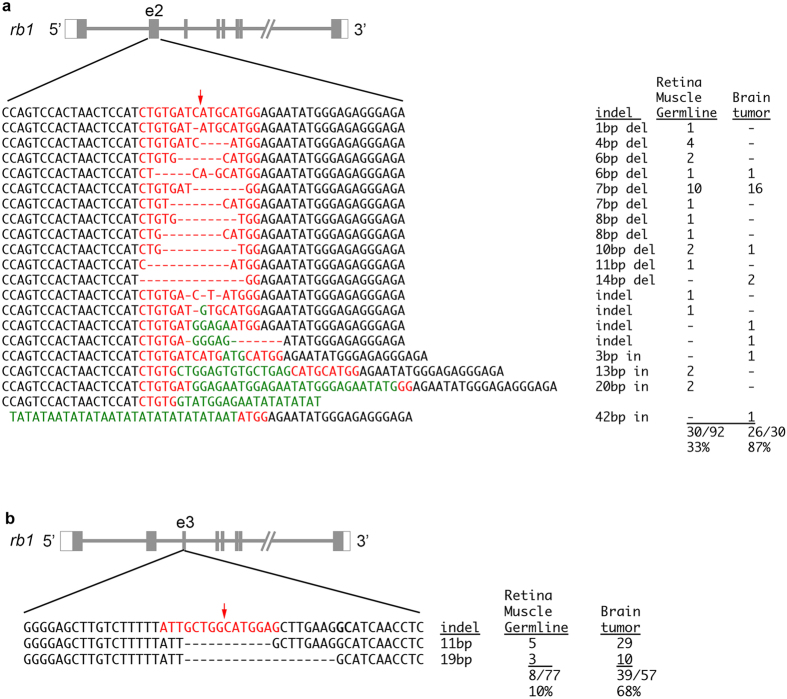
*rb1* mutant alleles and allele frequencies in somatic, germline, and tumor tissues from adult genetic mosaic zebrafish. (**a**) 14 different indel alleles in *rb1* exon 2 were identified in retina, muscle, and germline genomic DNA from two adults. Mutant alleles represented 33% of cloned amplicons. In tumor tissue 8 unique alleles were identified, representing 87% of cloned amplicons. (**b**) Two frameshift alleles that delete 11 bp and 19 bp in *rb1* exon 3 were detected in two adults. 10% of sequenced clones were mutant in retina, muscle or germline tissue. In tumor tissue 68% of sequenced clones were mutant alleles. Red arrows mark TALEN cut site.

The frequency of *rb1* mutant alleles in tumor and adult tissues was examined to confirm that *rb1* somatic inactivation correlated with brain tumor induction in adult fish (Fig. 4). Targeted *rb1* exons 2 and 3 were sequenced in tumor, retina, muscle and germline tissue from four tumor positive fish ranging in age from 3.5 months to one year. In all tumor tissues (4/4) the frequency of *rb1* mutant alleles was consistently higher than wild type alleles (68%–87% of cloned amplicons represented indels; Supplementary Table 1). Only one or two specific *rb1* alleles were present in 3 out of 4 tumors. Of the 10 alleles recovered in the tumor tissue, 8 of the 10 were frameshift mutations. In contrast, the mutation frequency in retinal, muscle, and germline tissues was highly variable (3%–100%) and was as high as 7 different mutant *rb1* alleles in one fish (Supplementary Table 1). The presence of predominantly loss of function alleles in genetic mosaic fish that develop tumors is consistent with biallelic inactivation of *rb1* initiating the pathway to transformation. However, the possibility that additional mutations in the genome caused by off targeting contributed to tumor induction cannot be ruled out. The high efficiency of TALENs targeting *rb1* exon 2 produced 19 different indels, with five specific to tumor tissue (Fig. 4a). Remarkably, only two exon 3 frameshift alleles, Δ11 and Δ19, were recovered in the normal and tumor tissues from fish targeted with the low efficiency exon 3 TALENs (Fig. 4b). The high degree of mutagenesis was consistent with tumor growth from a neoplastic cell population transformed by inactivation of *rb1*.

In summary, we demonstrate that targeted somatic inactivation of the *rb1* tumor suppressor in zebrafish embryos using two independent TALEN pairs results in genetic mosaic adults that develop tumors at high frequency, predominantly located in the brain. Pathological and immunohistochemical analyses of the head in affected individuals indicate zebrafish can be predisposed to neuroectodermal-like and glial-like tumors by somatic inactivation of *rb1* with site-specific nucleases. In contrast to genetic mosaic animals, adult *rb1*–/+ heterozygotes with a stable germline *rb1* mutation do not develop tumors. This suggests that nuclease targeting induces biallelic inactivation at the *rb1* locus and sensitizes cells to transformation. It has been proposed that in the case of epithelial cancers, tumor initiating cells may arise from a dedifferentiating progenitor population of transit amplifying cells^29^. Similar mechanisms may drive neoplasms derived from the neurogenic cell populations in the brain. In support of this, mouse models of pediatric and adult brain tumors have been created by targeting tumor suppressor gene knockout, oncogene activation and insertional transposon mutagenesis to neural stem and progenitor cells in the brain^21^,^22^,^23^,^24^,^25^,^26^,^30^,^31^,^32^,^33^. Our observations are consistent with previously published zebrafish models of brain cancer generated by driving overexpression of activated Ras^34^,^35^ in neural and glial progenitor cells. Adapting a tissue specific CRISPR targeting method^6^ to inactivate genes in neural progenitors in combination with zebrafish brain cancer models^35^,^36^,^37^ will be useful for investigating cooperating pathways promoting tumorigenesis. Our study demonstrates the potential for rapid large scale screening of candidate tumor suppressors in zebrafish using genome-editing nucleases to generate genetic mosaics.

## Methods

### Zebrafish care and husbandry

Zebrafish were reared in an Aquatic Habitat system from Aquatic Ecosystems, Inc., and maintained at 27 ^o^C on a 14 hr light/10 hr dark cycle. The WIK wild type strain used in this study was obtained from the Zebrafish International Research Center (http://zebrafish.org/zirc/home/guide.php). Experimental protocols were approved by the Iowa State University Institutional Animal Care and Use Committee (Log # 11-06-6252-I). The protocols are in compliance with American Veterinary Medical Association and the National Institutes of Health guidelines for the humane use of laboratory animals in research. Adult fish were anesthetized and euthanized in MS-222 Tricaine Methanesulfonate, and tissue dissection was carried out according to experimental protocols approved by the Iowa State University Institutional Animal Care and Use Committee (Log # 11-06-6252-I) in compliance with the American Veterinary Medical Association and the National Institutes of Health guidelines for the humane use of laboratory animals in research. NIH/Office of Animal Care and Use/Animal Research Advisory committee (ARAC) Guidelines for endpoint in neoplasia studies (oacu.od.nih.gov/ARAC/Guidelines for Endpoints in Animal Study Proposals) were used to establish a humane endpoint in the zebrafish *rb1* TALEN injected fish. Adult fish were monitored for general appearance, size, length, viability and morbidity relative to control siblings daily during routine feeding. Fish were closely monitored bi-weekly for gross presentation of cranial tumors. Fish from each injection experiment were sacrificed before tumor burden reached 3 mm in size/25 mg in weight, constituting less than 10% of the total body weight of an adult fish (300–500 mg), as outlined for mouse and rat studies^38^, or by 1.5 year of age, whichever endpoint was reached first. The swimming behavior of some *rb1* TALEN injected fish was adversely affected before gross presentation of cranial tumors, possibly due to tumor location. These fish were sacrificed although tumor burden was less than 3 mm/10% of body weight.

### rb1 TALEN assembly, targeting, and allele analysis

TALENs targeting *rb1* exon 2 or exon 3 were designed using TAL Effector Nucleotide Targeter 2.0 at https://tale-nt.cac.cornell.edu/node/add/talen-old^39^ and assembled in the modified GoldyTALEN scaffold^5^. 150–200 pg of exon 2 or 12.5–50 pg of exon 3 TALEN mRNA was injected into 1-cell stage wild type WIK embryos. TALEN efficiency was determined by assaying individual injected embryos for disruption of restriction enzyme sites in a PCR amplicon spanning the target site. Genomic DNA was extracted from embryos and adult fin clips by placing tissue in 50 μl 50 mM NaOH and heating at 95 ^o^C for 30 minutes. For *rb1* allele frequency analysis, genomic DNA was isolated from tumor, somatic and germline tissues using a DNeasy Blood and Tissue Kit (Qiagen). Amplicons were TOPO cloned (Invitrogen), and 50–100 individual clones sequenced per sample. Primers used for amplification of *rb1* exons: exon 2 F-5’- TTTCCAGACACAAGGACAAGG-3’, R-5’-GCGGTAAAGCAGATATCAGAAGA -3’; exon 3 F-5’-TTTCCAGACACAAGGACAAGG-3’, R-5’-CCTCACAAGACTGATGAACTGC-3’ or F-5’-TTACCGCACTTGTGTTTTGG-3’, R-5’-TGCCACACATACCTCAGACC-3’.

### Histopathology and Immunohistochemistry

Adult zebrafish were sacrificed and head tissue processed for histopathology and immunohistochemistry as described previously^40^. For histopathology tissues fixed in Davidson’s or 10% Formalin (Fisher) were decalcified in Cal-Ex (Fisher), and paraffin embedded at the Iowa State University Clinical Histopathology Laboratory. 6 um sections were stained with Hematoxylin 7211 Richard-Allan Scientific (Fisher) and 3% Eosin Y (Argos Organics). For immunohistochemistry heads were fixed in 4% paraformaldehyde, decalcified in 12% EDTA, and embedded in Tissue-Tek optimal cutting temperature medium (Sakura). 14 um tissue sections were labeled with antibodies anti-phosphohistone-H3 (1:100, Upstate), anti-S100 (1:250, Dako). Secondary antibodies were conjugated with HRP. Tissues were counterstained with modified Mayer’s Hematoxylin (Fisher) and eosin. Slides were photographed on a Zeiss Axiophot using a Nikon Rebel camera and on a Zeiss confocal LSM 700.

## Additional Information

**How to cite this article**: Solin, S. L. *et al.* Rapid tumor induction in zebrafish by TALEN-mediated somatic inactivation of the *retinoblastoma1* tumor suppressor *rb1*. *Sci. Rep.* **5**, 13745; doi: 10.1038/srep13745 (2015).

## Supplementary Material

Supplementary Information

## Figures and Tables

**Table 1:** Tumor frequency and location in adult zebrafish after somatic inactivation of *rb1*.

TALEN-targeted *rb1* exon	Tumor number andfrequency (%)^a^	Tumor location andhistological features	Number
Exon 2^b^	29/87 (33%)	Brain	23/29
		glial or glial/neuronal	1
		expanded glial rests	2
		neuroectodermal-like	5
		not analyzed by histology^c^	15
		Unknown^d^	6/29
Exon 3^e^	29/275 (11%)	Brain	29/29
		glial or glial/neuronal	3
		neuroectodermal-like	3
		not analyzed by histology^c^	23
Control un-injected^f^	0/150	–	–

^a^Frequency and percentage of adult fish with tumors at 1.5 years of age.

^b^Four separate injections of 150 pg or 200 pg.

^c^Adults presenting with gross tumors in head region; morphology of dissected brain was abnormal and disrupted by mass lesion; histopathology not performed.

^d^Tumors located in head region; dissection and histopathology not performed.

^e^Three separate injections of 12.5, 25, or 50 pg.

^f^Control un-injected fish raised alongside TALEN-injected embryos.
