# Supplementary material for: Rapid tumor induction in zebrafish by TALEN-mediated somatic inactivation of the retinoblastoma1 tumor suppressor rb1
Source: Sci Rep. 2015 Sep 8;5:13745. doi: 10.1038/srep13745 (PMC4642565; doi:10.1038/srep13745)
Supplement: Supplementary Information [file srep13745-s1.pdf]

# Rapid tumor induction in zebrafish by TALEN-mediated somatic inactivation of the retinoblastoma1 tumor suppressor *rb1*

Staci L. Solin[1], Heather R. Shive[2], Kevin D. Woolard[3], Jeffrey J. Essner[1], Maura McGrail[1]*

Supplementary Information

**Supplementary Figure S1.** Design of TALENs targeting exon 2 and exon 3 of zebrafish *rb1*. a, Diagram of TALEN design to target *rb1* exon 2. Gel of restriction enzyme assay on exon 2 PCR amplicon demonstrates 100% biallelic inactivation at the target site in 50% of individual embryos injected with 150 pg TALEN mRNA. b, Diagram of TALEN design to target *rb1* exon 3. Gel of restriction enzyme assay on exon 3 PCR amplicon reveals relatively inefficient inactivation of the targeted location in individual embryos injected with 50 pg TALEN mRNA. Higher doses of exon 3 TALENs were not used due to toxicity. c, Four new germline mutations recovered in *rb1* exon2. Two frameshift and two inframe deletion alleles have been established.

**a**

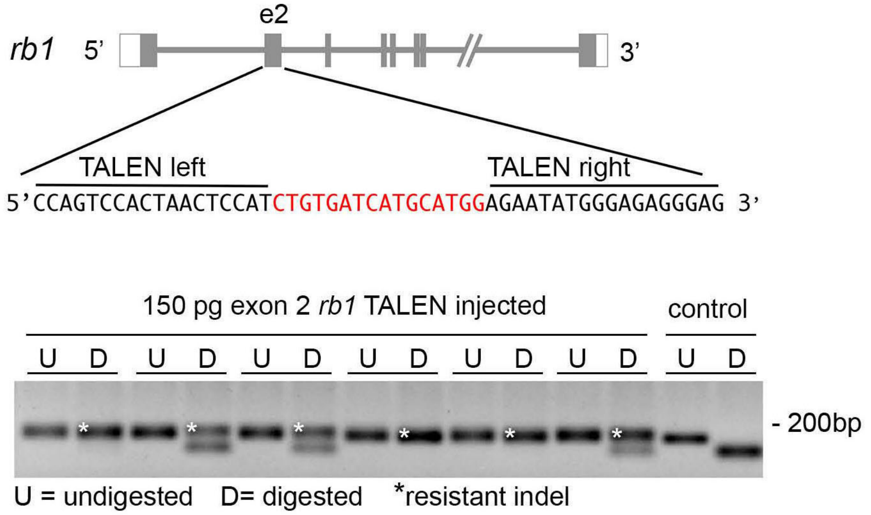


**b**

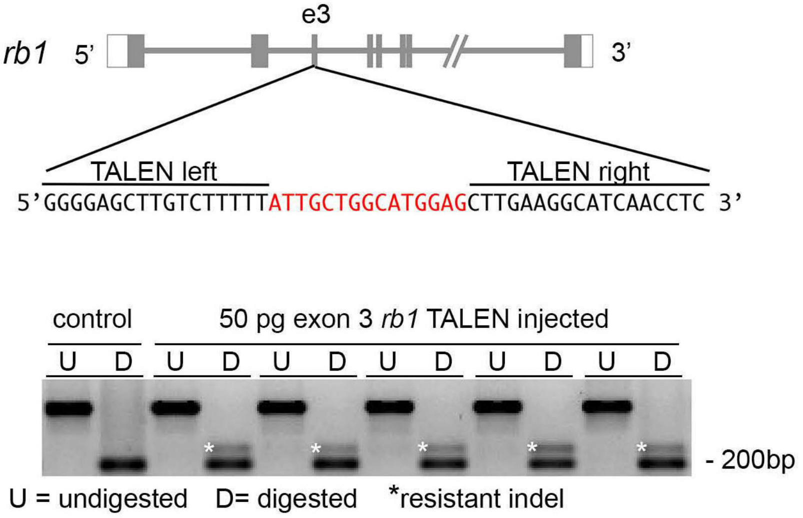


**c** recovered germline mutations in *rb1*

```
5'CCAGTCCACTAACTCCATCTGTGATCATGCATGGAGAATATGGGAGAGGGAGA3' WT
5'CCAGTCCACTAACTCCATCTGTG----TGCATGGAGAATATGGGAGAGGGAGA3' Δ4
5'CCAGTCCACTAACTCCATCTGTGA-------TGGAGAATATGGGAGAGGGAGA3' Δ7
5'CCAGTCCACTAACTCCATCTGTGA---------GAGAATATGGGAGAGGGAGA3' Δ9
5'CCAGTCCACTAACTCCATCTGTGAT------------ATATGGGAGAGGGAGA3' Δ12
```

**Supplementary Figure S2.** Regions of high mitotic activity in *rb1* inactivation-induced brain tumors. Coronal sections through wild type (a, b) two *rb1* TALEN exon 2-induced tumors (c-f) and one *rb1* TALEN exon 3-induced tumor (g,h). H and E staining reveals presence of tumor and disruption of brain architecture. Phospho-histone pH3 labeling reveals absence of pH3 labeling in wild type brain (b) compared to regions with high mitotic index in the three tumors (d, f, h). Scale bars in H&E panels 500 um; pH3 panels 50 um, inset 20 um.

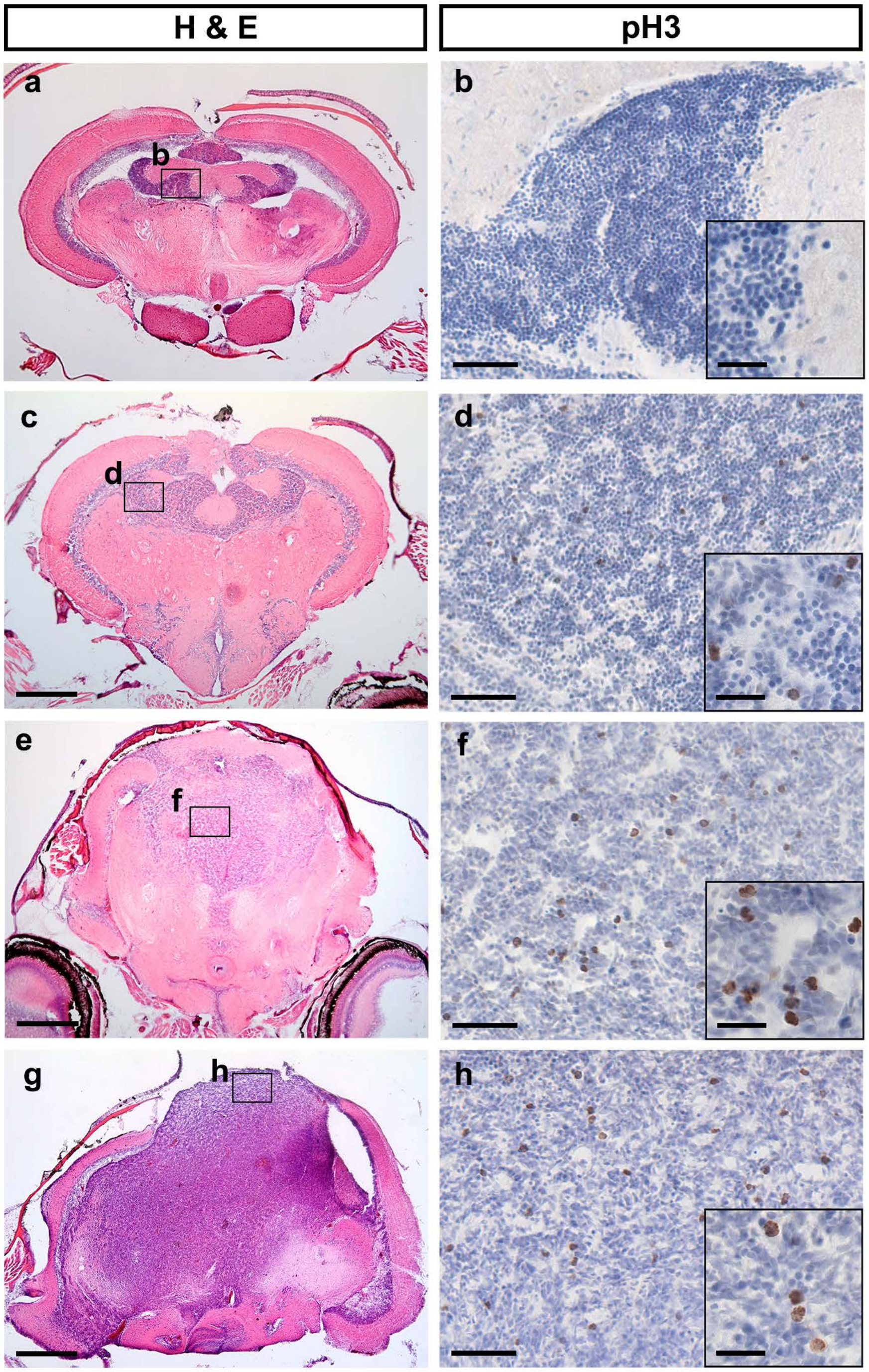

**Supplemental Table S1.** *rb1* genetic mosaicism in adult tissues of TALEN targeted embryos

| Fish ID | Tumor | | Retina | | Muscle | | Germline[a] | |
|---|---|---|---|---|---|---|---|---|
| | frequency[b] | alleles[c] | frequency | alleles | frequency | alleles | frequency | alleles |
| Exon 2 -1 | 13/15 (87%) | 1 | 2/16 (13%) | 2 | 9/12 (75%) | 7 | 10/10 (100%) | 4 |
| Exon 2 -2 | 10/16 (63%) | 7 | 3/16 (19%) | 3 | 6/22 (27%) | 5 | 1/19 (5%) | 1 |
| Exon 3-1 | 17/21 (81%) | 1 | 4/30 (13%) | 2 | 2/22 (9%) | 2 | 2/27 (7%) | 2 |
| Exon 3-2 | 22/36 (61%) | 2 | 0/20 (0%) | - | 1/27 (4%) | 1 | 1/29 (3%) | 1 |

[a] Ovary or Testis. [b] Frequency of insertion/deletion alleles in cloned amplicons from genomic DNA. The number of indel amplicons out of total amplicons sequenced is shown. [c] Number of unique mutant alleles.
